# Economic evaluation of percutaneous cryoablation vs conventional surgery in extra-abdominal desmoid tumours in the Spanish healthcare system

**DOI:** 10.1186/s13244-023-01580-w

**Published:** 2024-01-07

**Authors:** José Antonio Narvaez, Daniel Bernabeu, Lorenzo Muntaner, Fernando Gomez, José Martel, María del Mar Castellano, Raúl García-Marcos, Reda Britel, Itziar Oyagüez, Nerea Tejado, Eduardo Ortiz-Cruz

**Affiliations:** 1 Musculoskeletal Radiology, Hospital Universitario de Bellvitge-IDIBELL, Barcelona, Spain; 2 Faculty of Medicine and Health Sciences, Universitat de Barcelona, Barcelona, Spain; 3 Musculoskeletal Radiology, Hospital Universitario La Paz, Madrid, Spain; 4 Hospital Son Espases, Radiología Intervencionista, Mallorca, Spain; 5 Hospital Sant Joan de Deu, Radiología Intervencionista, Barcelona, Spain; 6 Departamento de Radiología Musculoesqueletica, Hospital Universitario Fundación Alcorcón, Madrid, Spain; 7 Hospital Virgen de Las Nieves, Radiología Intervencionista, Granada, Spain; 8 Radiología Intervencionista, Hospital Universitario La Fe, Valencia, Spain; 9 Pharmacoeconomics & Outcomes Research Iberia (PORIB), Madrid, Spain; 10 Cirugía Ortopédica Oncológica, Hospital Universitario La Paz, Madrid, Spain

**Keywords:** Cryotherapy, Ablation techniques, Desmoid-type fibromatosis, Economic evaluation, Surgery

## Abstract

**Background:**

Desmoid tumours (DTs) or deep fibromatosis are benign soft-tissue tumours, sometimes locally aggressive, requiring intervention on some cases. Surgery has been the gold standard, but new less invasive techniques such as percutaneous cryoablation have proved their effectiveness, reducing health resources and complications. The study aimed to compare the total cost of percutaneous cryoablation and conventional surgery for patients with extra-abdominal and/or abdominal wall DTs, candidates for local ablative treatment in Spain.

**Methods:**

A cost-analysis model was developed. An expert panel provided data about resource consumption for the percutaneous cryoablation technique and validated the epidemiology used for target population estimation. Unitary resources cost (€ 2022) derived from local cost databases. A retrospective analysis of 54 surgical cases in 3 Spanish hospitals was performed to estimate the cost of conventional surgery based on the cost of the Diagnosis-Related group (DRG) codes identified on this patient sample, weighted by each DRG proportion. The total cost for each alternative included intervention cost and complications cost, considering debridement required in 4.5% of cases with percutaneous cryoablation and minor surgery for surgical site infection in 18.0% for conventional surgery.

**Results:**

The total cost for percutaneous cryoablation (€ 5774.78/patient-year) was lower than the total cost for conventional surgery (€ 6780.98/patient-year), yielding cost savings up to € 80,002 in 1 year for the entire cohort of 80 patients with DTs eligible for intervention estimated in Spain. One-way sensitivity analyses confirmed the results’ robustness.

**Conclusion:**

Percutaneous cryoablation versus conventional surgery would yield cost savings for the management of DT patients in Spain.

**Critical relevance statement:**

This manuscript provides insight into the economic impact derived from the savings related to the use of percutaneous cryoablation for desmoid-type tumours from the perspective of the Spanish National Healthcare System, providing useful information for the health decision-making process.

**Key points:**

• Desmoid tumours are locally aggressive and may require local therapy.

• Percutaneous cryoablation procedure is less invasive than the conventional surgery.

• Cost comparison shows savings associated to percutaneous cryoablation use.

**Graphical Abstract:**

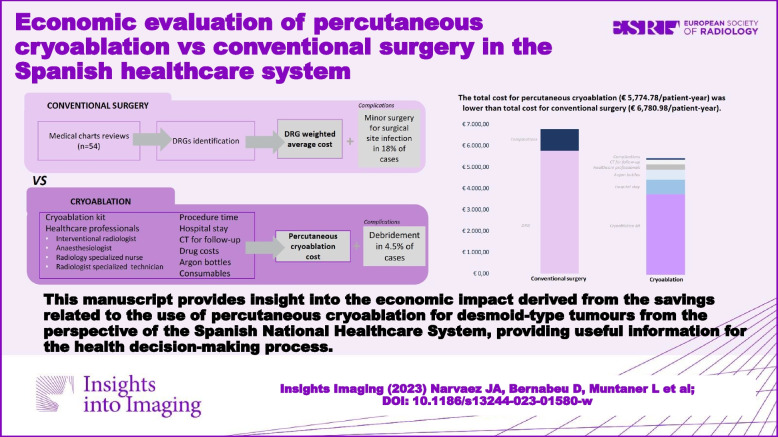

## Introduction

Desmoid tumours (DTs), also named desmoid-type or deep fibromatosis, are benign tumours that originate in musculoaponeurotic structures without malignant or metastatic potential but are locally infiltrating and usually recur after treatment [1, 2]. The World Health Organization has classified DTs as intermediate, locally aggressive tumours [3]. DTs represent less than 3% of soft tissue neoplasms and less than 0.03% of all tumours, with an annual incidence between 2 and 4 cases/million patients/year [1, 4]. DTs have a higher prevalence in women (2:1) [5], and their frequency increases in the 3rd and 4th decade of life. In some patients, DTs can be locally aggressive, causing pain, disability and exceptionally death, especially in the intra-abdominal area [1]. Although DTs can be located in any area of the body they are most frequently found (35–50%) in the intra-abdominal area (usually associated with Gardner syndrome). Shoulder girdle, chest wall and inguinal regions are among the most prevalent extra-abdominal sites [6–8].

The management of DTs has evolved in recent years, but there is still no clear consensus about the best treatment option for DTs [2, 9]. In the management and treatment guidelines recently published by the *Desmoid Tumour Working Group* (DTWG), active tumour surveillance is recommended as the first option [10], although conventional surgery continues to be strategy most used in clinical practice, specifically in the case of extra-abdominal DTs [9], and also as one of the main alternatives in cases of progressive or symptomatic disease, along with systemic treatment or radiotherapy [2]. Surgery can be effective for achieving local control of the tumour, though it can lead to certain drawbacks, such as a high rate of recurrence up to 40% (exceeding published and acceptable local recurrence rates), which may increase up to 90% after a first relapse [11], neurovascular damage, potential activation of residual tumours, and long postsurgical recovery [8].

The intervention chosen to treat DTs depends on the location of the tumour. The DTWG recommendations mention that local ablative treatments such as cryotherapy or radiotherapy are options that can be considered as an alternative to medical therapies on an individual basis [10]. The European Society of Medical Oncology (ESMO) guidelines [12] establish a similar approach for progressing cases, which needs to be individualised on a multidisciplinary basis from watchful waiting, systemic therapies, or local therapies such as percutaneous cryoablation for extra-abdominal cases, to achieve the optimal strategy.

In this complex clinical scenario, the use of percutaneous cryoablation in progressed extra-abdominal DTs has recently increased and displayed promising results [1, 2, 4, 8, 13–16].

Percutaneous cryoablation is a minimally invasive technique that causes cell death by freezing the affected tissue [17, 18]. It is a safe intervention since it minimises the damage to the collagen structures that surround the tumour [19]. In musculoskeletal lesions, percutaneous cryoablation can play an important role in patients with benign tumours or metastases, either for palliative purposes or for local tumour control [20]. In addition, it is associated with low morbidity and can be repeated if necessary [19]. In primary or locally recurrent extra-abdominal DTs, percutaneous cryoablation prevents primary local recurrence and achieves long-term disease control comparable to surgery [15]. Compared to surgery, percutaneous cryoablation is less invasive, requires shorter anaesthesia times, has a lower economic impact, and does not require the use of an operating room [21]. On the other hand, conventional surgery is correlated with a higher risk and morbidity, lower quality of life, and longer hospital stays, which generate higher costs for the healthcare system [20].

With new health technologies entering the market an economic appraisal it is required to allocate wisely the available resources. From an economic point of view, there have been no studies that quantify and compare the costs of the interventions commonly used to treat DTs at a national level.

To the best of the authors’ knowledge, this is the pioneering study evaluating the cost of percutaneous cryoablation versus conventional surgery for patients with extra-abdominal desmoid tumours in Spain. The analysis yields valuable findings for informed health decision-making.

## Materials and methods

A cost analysis model was developed in Microsoft Excel® (version 2308 including in Microsoft 365) to estimate and compare the total costs associated with percutaneous cryoablation and conventional surgery for DTs management.

The population assessed in the present analysis refer to patients with DTs located in the extra-abdominal area and/or abdominal wall who were candidates for the percutaneous cryoablation technique or conventional surgery in Spain. Cases of desmoid fibromatosis located in the retroperitoneum or in the abdominal cavity were not considered and were excluded from the analysis.

The results of the analysis are expressed in total costs of percutaneous cryoablation, total costs of conventional surgery, and the difference in costs between the two interventions. The costs were estimated per patient and for the entire study cohort.

### Perspective and time horizon

The analysis was carried out from a hospital perspective, therefore, only the direct hospital healthcare costs were considered. Indirect costs and social costs were excluded from the analysis. The model utilised a time horizon of 1 year, so not discount rate was applied.

### Advisory board

All the inputs (resource consumption and cost) used in the model were provided and validated by a panel composed of eight clinical experts whose main areas of specialisation were interventional radiology and orthopaedic oncology surgery.

A structured questionnaire that included the values identified in the scientific published literature was designed and individually filled by each of the experts. Subsequently, an expert meeting was carried out to validate and agree upon all the values used in the analysis. In the consensus process, experts made decisions by agreement, working together to find a mutually acceptable value.

### Estimation of the target population

**Fig. 1:**
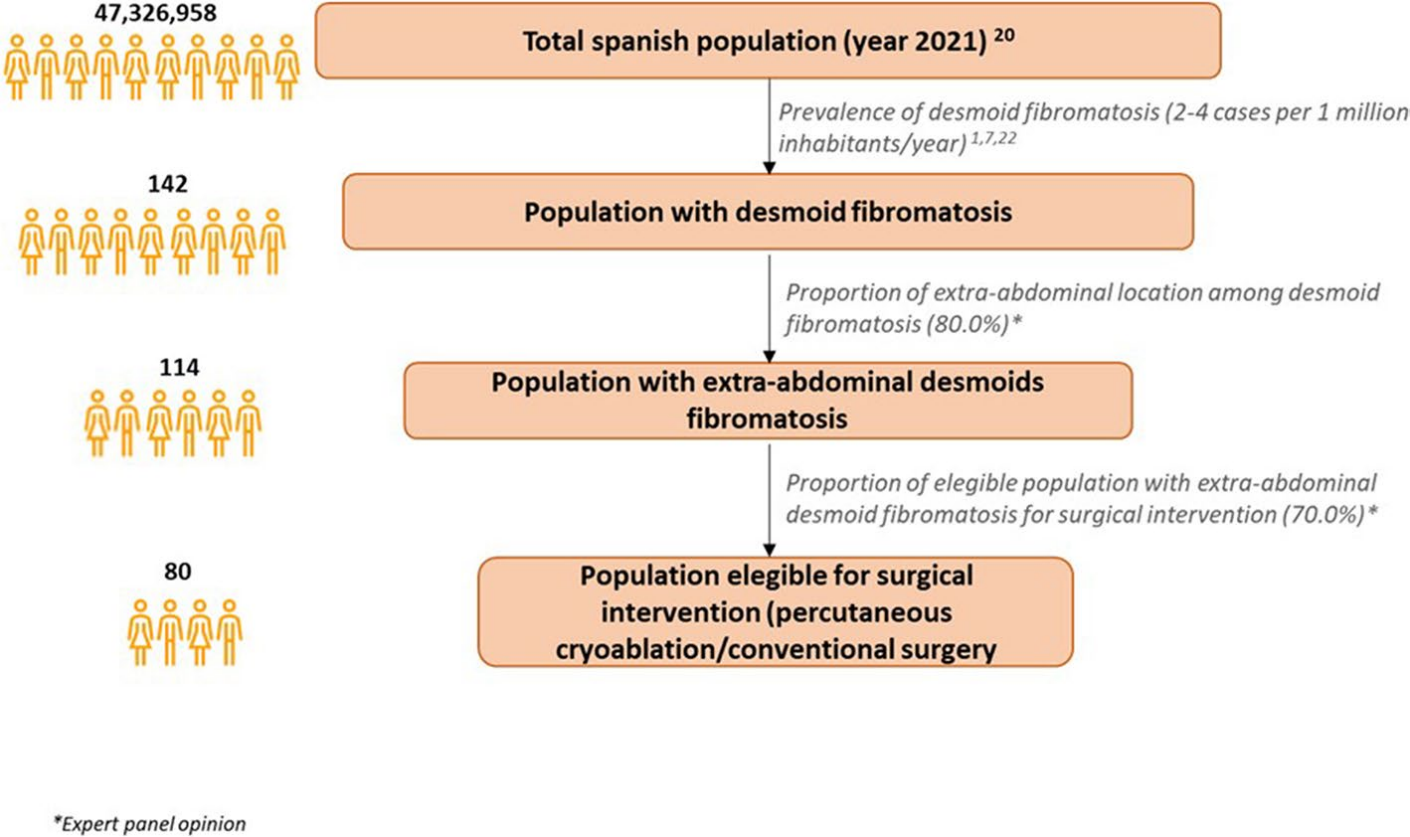
Estimation of the target population

Figure 1 shows the epidemiological flow used to estimate the target population referred to the population with DTs eligible for intervention (surgery or percutaneous cryoablation). To calculate the total cohort size of patients in the analysis, the projections of the total population residing in Spain in 2021 published in the Portal of the National Institute of Statistics were used [22]. Thereafter, the annual incidence data of 2–4 cases of DTs per million inhabitants, reported by various studies, were applied [1, 7, 23]. Next, it was agreed by the members of the expert panel that 80% of DTs were located in the extra-abdominal area and/or in the abdominal wall, of which 70% could be subject to percutaneous cryoablation or surgery.

### Percutaneous cryoablation

The estimation of the total cost for percutaneous cryoablation was calculated by the sum of the costs derived from the procedure and technique and cost associated to complications management.

The resources required for percutaneous cryoablation procedures (cryoablation probes, health professionals, imaging techniques for procedure follow-up, medications and consumables) were identified and validated by the expert panel. The consumption of each of the resources were also provided and/or validated by the advisory board (Table 1). Unitary costs extracted from national databases [24, 25] and expressed in 2022 euros, were used for calculation of total cost of percutaneous cryoablation technique and procedure.

**Table 1:** Resource consumption and unitary cost associated to cryoablation

Resource	Units	Unitary cost (€ 2022)
Cryoablation kit	1*	€ 3993.00 (VAT included-21%) ^Ŧ^
Healthcare professionals		
*Interventional radiologist*	2*	€ 0.38/min [24]
*Anaesthesiologist*	1*	€ 0.38/min [24]
*Radiology specialised nurse*	1*	€ 0.22/min [24]
*Radiologist specialised technician*	1*	€ 0.17/min [24]
Procedure time	173 min^7^	
Hospital stay (days)	1*	€ 711.81 per day [23]
Computed tomography for follow-up	1^2,14,16^	€ 239.41 [23]
Drugs		€0.77
*Fentanyl*	180.00 mg**	€ 4.3520/mg ^ŦŦ^
*Propofol*	322.50 mg**	€ 0.000002/mg ^ŦŦ^
*Midazolam*	2.55 mg**	€ 0.000155/mg ^ŦŦ^
*Paracetamol*	1000.00 mg**	€ 0.000000/mg ^ŦŦ^
*Dexketoprofen*	50.00 mg**	€ 0.000053/mg ^ŦŦ^
Argon bottles (number)	2*	€ 247.25*
Consumables		€ 43.24
*Sterile gauze*	8*	€ 0.08 [26]
*Sterile drapes*	4*	€ 0.57 [27]
*Surgical gloves (pair)*	5.5*	€ 0.25 [27]
*Scalpel blade*	1*	€ 0.18 [27]
*Catheters for dissection*	0.5*	€ 0.06 [27]
*Needles*	3*	€ 1.89 [27]
*Saline solution (500 ml)*	3.5*	€ 1.08 [27]
*Syringes*	2.5*	€ 0.04 [27]
*CO*_*2*_* medicinal (bottle 5L)*	1*	€ 29.19 [27]
Complications		
*Debridement*		€ 614.94 [23]
*Surgery (minor severity)*		€ 5680.01 [23]

^*^Expert panel. **Bellvitge Hospital Protocol.^Ŧ^ Boston Scientific. ^ŦŦ^ Botplus 2.0,2022

The use of a percutaneous cryoablation kit (Boston Scientific®) was defined per procedure and patient [4]. A cryoablation kit, which includes 5 sterile, 17-gauge cryoablation probes — Icesphere, IceRod (Boston Scientific®) — was considered enough to treat most cases, based on data collected in a recent systematic literature review and meta-analysis of percutaneous cryotherapy of extra-abdominal DTs [28], and expert panel agreement.

The participation of the following health professionals was considered to be required for the performance of the intervention: two interventional radiologists (area medical specialists), one anaesthesiologist, one radiology nurse, and one radiology technologist, with an approximate total time dedication of 173 min. Hospitalisation of one night was also considered after intervention for observation previously to the patient hospital discharge [8, 18], and computed tomography was considered the imaging technique for monitoring the intervention, according to the evidence and the expert panel agreement [2, 15, 19].

Conscious sedation and local anaesthesia were considered required for percutaneous cryoablation. The anaesthetics and analgesics used in most procedures were fentanyl, propofol, midazolam, paracetamol, and dexketoprofen.

Current cryoablation systems use argon, which circulates through fine probes to induce rapid freezing and thawing of tissues [2, 21]. It was determined that two bottles of argon were required per patient and procedure. It was assumed that the equipment required for the intervention (cryoablation system) was leased, so no costs were imputed in this area. In addition, many consumables used during the procedure were counted: sterile gauze, sterile drapes, scalpel blades, catheters for dissection, etc. (Table 1).

In addition to quantifying the costs of the percutaneous cryoablation procedure, the possible complications derived from the intervention were also evaluated. Based on the literature and validated by the expert panel, a complication rate of 4.5% was established, with the most frequent complication being cutaneous necrosis treated with debridement [4, 15] (Table 1).

### Conventional surgery

The analysis of cost of surgical of extra-abdominal DTs is difficult, and no studies reporting the cost of surgery on DTs were identified, but the Diagnosis-Related Group (DRG) system of payment may be a useful tool. This system is a kind of patient classification system that divides patients into different groups according to their age, whether a medical intervention is performed, and whether there are comorbidities or complications. When a hospital treats a patient in a certain DGR category, the fee paid to the provider is fixed, regardless of the actual medical expenditure. The DRG payment method has been successfully implemented in the United States, Germany, and other countries, and it is currently recognised internationally as a better payment method [29, 30].

In the present analysis, an approximation was made using the costs of DRGs, in its most current version, All Patient Refined Diagnosis-Related Groups (APR-DRG). As not specific DRG is available for DT surgery, a retrospective analysis of the clinical history of cases of extra-abdominal DTs undergoing surgery in three Spanish teaching hospitals: Bellvitge University Hospital (Barcelona), La Paz University Hospital (Madrid), and Son Espases University Hospital (Balearic Islands) was performed to identify the APR-DRG associated with each case. We applied a protocol for a multicentre retrospective observational study that was evaluated and approved by the Clinical Research Ethics Committee of the Bellvitge University Hospital (code PR075/22). The following inclusion case selection criteria were established: patients with DTs located in the extra-abdominal and/or abdominal wall area who underwent surgery from 2010 to 2021 and who would have been candidates for percutaneous cryoablation. The Statistical Portal of the Spanish Ministry of Health was used to obtain the unit costs of the identified DRGs [31], in the autonomous regions of the aforementioned hospitals. Finally, the total cost of the conventional surgical process was calculated by applying a weighting to each DRG identified (93, 98, 227, 312, 317, 320, 343, 351, 362, 532, 680 and 951) (Table 2) as a function of the total number of cases in these hospitals.

**Table 2:** Conventional surgery cost estimation

DRG	DRG description	Severity level	*N*	Proportion	Unitary cost* (€ 2022)	Partial cost
**93**	Sinus and mastoids procedures	2	1	1.9%	€ 4406.73	81.61
**98**	Other ear, nose, mouth and throat procedures	1	1	1.9%	€ 4995.09	92.50
**227**	Hernia procedures except inguinal and femoral	1	1	1.9%	€ 5782.12	107.08
**312**	Wound debridement and skin graft except hand for musculoskeletal and connective tissue disorders	1	2	3.7%	€ 9650.44	357.42
**317**	Soft tissue procedures	1	34	63.0%	€ 5027.38	3165.39
		2	5	9.3%	€ 7690.40	712.07
		3	2	3.7%	€ 12,863.95	476.44
**320**	Other musculoskeletal system and connective tissue procedures	2	1	1.9%	€ 5853.67	108,40
**343**	Pathological fractures and musculoskeletal and connective tissue malignancy	1	1	1.9%	€ 4679.89	86.66
**351**	Other musculoskeletal system and connective tissue diagnoses	1	2	3.7%	€ 3050.69	105.10
**363**	Breast procedures except mastectomy	1	1	1.9%	€ 5675.23	58.92
**532**	Menstrual and other female reproductive system disorder	2	1	1.9%	€ 3181.56	173.25
**680**	Myeloproliferative disorders or poorly differentiated neoplasm with major OR procedure	1	1	1,9%	€ 9355.60	112.99
**951**	Extensive or procedure unrelated to principal diagnosis	1	1	1.9%	€ 6520.64	120.75
	**Total**		54	**100%**	-	**€ 5758.58**

^*^Average cost for acute hospitalisations on Statistical website of National Health Ministry, corresponding to Madrid, Catalonia and Balearic Islands

Regarding complications associated with surgery, surgical site infection treated with further surgery (minor severity) was stipulated as the main complication, with a rate of 18.0% [32].

### Sensitivity analysis

One-way sensitivity analyses were carried out to test the model robustness. Exploratory the values of the following parameters were individually modified (increased or decreased) by ± 10%: prevalence (cases/million), proportion of patients with DTs in the extra-abdominal area, and the proportion of patients suitable for percutaneous cryoablation or surgery; for the percutaneous cryoablation alternative- the time (min) of the procedure, the stay (days), the proportion of patients who would require debridement, the unit costs of the cryoprobes kit, the cost of the debridement procedure, the daily cost of the hospital stay, and the cost of the argon; for conventional surgery, the proportion of patients who would require minor surgery to manage the complication of infection, the unit cost of said surgery, and the unit cost of the surgical procedure estimated from the DRGs.

Additionally, a complication rate of 20.8% for conventional surgery from an alternative source [33] was also tested.

A total of 27 potential scenarios derived from the individual modification of the value of each parameter were assessed, to identify the parameters with the higher influence on the results.

## Results

The estimation of the target population of the analysis was carried out based on the epidemiological data described in Fig. 1. A total cohort of 80 patients with DTs eligible for intervention was calculated.

The retrospective analysis of the surgical cases in the three Spanish hospitals comprised 54 patients with an average age of 47 years (± 18.4), 59.4% were females, with the following distribution according to DT localisation: 37.0% at the abdominal wall, 35.2% at body trunk, 20.4% at extremities and 7.4% at head and neck. The average size of lesions 6.6 cm (range:1.1–15 cm) was estimated with the maximum diameter reported. Most of the cases (75.9%) corresponded to APR-DRG 317 (Soft tissue procedures). An average unit cost of € 6780.98 per patient undergoing conventional surgery was obtained, derived from the unit cost of the identified APR-DRGs weighted by the proportion of cases detailed in Table 2.

In the base case, percutaneous cryoablation generated a total cost of € 5774.78 per patient per year and € 459,149 for the entire cohort included in the analysis for 1 year. In the case of conventional surgery, the total cost per patient and year rose to € 6780.98, which translated to € 539,151 for the whole population with DTs in Spain in 1 year. Consequently, when comparing percutaneous cryoablation against conventional surgery, an incremental cost of € − 1006.20 per patient per year was obtained, which is equivalent to total cost savings for the Spanish National Health System (NHS) up to € 80,002 per year for the 80 patients included in the target population (Table 3).

**Table 3:** Base case analysis results

	**Total cost per patient**	**Total cost for the whole patient cohort**
**Percutaneous cryoablation**		
Cryoablation kit	€ 3993.00	€ 319,440.00
Healthcare professionals	€ 264.38	€ 21,150.40
Hospitalisation	€ 711.81	€ 56,944.80
Computed tomography	€ 239.41	€ 19,152.80
Drugs	€ 0.77	€ 61.60
Argon	€ 494.50	€ 39,560.00
Consumables	€ 43.24	€ 3459.2
Complications	€ 27.67	€ 2213.78
**Conventional surgery**		
Surgery	€ 5758.58	€ 417,406.15
Complications	€ 1022.40	€ 72,815.20
**Incremental cost for cryoablation vs conventional surgery**		
	€ − 1006.20*	€ − 80,002.56*

^*^Negative quantities indicate savings (cost reduction) with percutaneous cryoablation against conventional surgery

The one-way sensitivity analysis results demonstrated the robustness of the model. Despite of the modifications tested around the 13 parameters, previously described, the use of percutaneous cryoablation compared to the conventional surgery remained being associated to cost savings for NHS in all the 27 scenarios.

The parameter with the highest impact on these savings was the unitary cost for conventional surgery estimated from DRG-APR. Percutaneous cryoablation could yield total annual savings for the NHS up to € 125,789 if conventional surgery cost would be € 6334/patient. On the other hand, a reduction of the conventional surgery cost to € 5759 would imply cost savings of € 34,216 with percutaneous Cryoablation use in the whole entire cohort of patients with DTs estimated for Spain.

The second most influential parameter was the unit cost of the cryoablation kit. Savings for NHS would range between € 111,751 considering a reduction of 10% in the cryoablation probes cost and € 48,254, considering an increase of 10% in this cost.

**Fig. 2:**
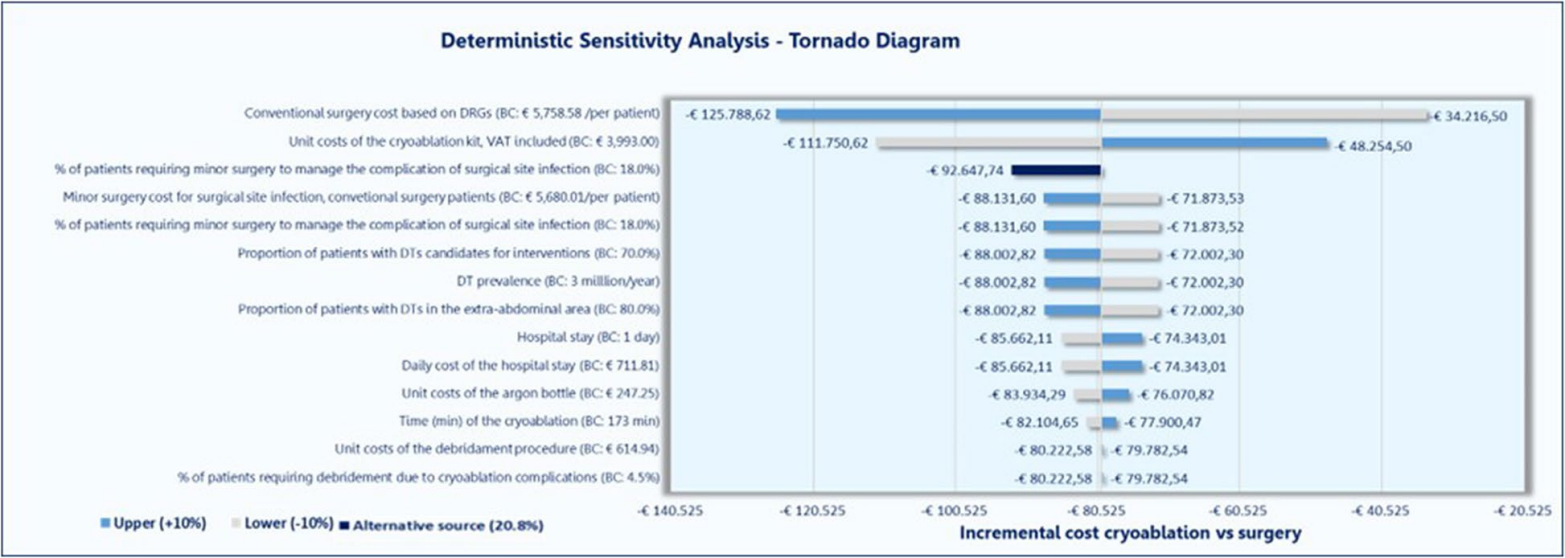
Tornado diagram for one-way sensitivity analysis results. DRG, Diagnosis-Related Groups

The modification of the values of the rest of the parameters did not generate relevant changes in the savings, as it is graphically shown in Fig. 2. Changes in the epidemiological data used for target population estimation would imply that annual cost savings oscillating ± € 8000 for the entire cohort of patients.

## Discussion

DTs are a rare type of benign musculoskeletal tumour, with a tendency to recur at the local level after treatment, but without the ability to cause metastasis, as indicated by the World Health Organization [3, 10]. DTs may behave aggressively, and when it progresses, it can lead to a loss of function in the affected area and pain, leading to a considerable deterioration in quality of life [34]. Standardising the management of this type of tumour is not easy, and numerous efforts have been made in recent years [10]. Among the main reasons are their unpredictable behaviour, which is difficult to predict at a biological level, and the wide range of therapeutic strategies to treat them [15]. Generally, the first therapeutic step consists of watchful waiting strategy or surveillance approach of the tumour; and in case the patient experiences progression or worsening of symptoms, interventional techniques are usually used [10]. Until recently, surgical resection was the standard of care for these patients [6, 35]; however, as part of the evolution of therapy away from primary surgical approaches to less invasive options, image-guided ablation has been accepted as less morbid and includes cryoablation, microwave and high-intensity focused ultrasound ablation [16, 36]. These less invasive techniques, such as percutaneous cryoablation, can avoid or reduce the level of morbidity associated to traditional surgery [15]. The management of asymptomatic or symptomatic patients with an initial watchful waiting approach or if local and/or systemic treatments are indicated, regardless of the site and size of the tumour, should be under the supervision of an expert multidisciplinary team.

A recent meta-analysis has concluded that percutaneous cryotherapy for extra-abdominal DT could be considered an option with equivalent efficacy to traditional strategies [28]. In that sense, in addition to being a technique with demonstrated efficacy and safety [37, 38], percutaneous cryoablation can lead to a lower consumption of hospital resources and lower rate complications and thus results in a lower economic impact than surgery [21]. In the present analysis, the possible savings that percutaneous cryoablation could generate compared to conventional surgery in the management of DTs in the Spanish hospital context have been highlighted: percutaneous cryoablation could avoid an expense of € 1000 per patient per year, which would mean annual savings of € 80,000 if this figure is extrapolated to the estimate of patients with DTs located in the extra-abdominal area and/or abdominal wall who are indicated for cryoablation and/or surgery in Spain.

Systemic medical treatments such as low-dose chemotherapy, hormonal agents, nonsteroidal anti-inflammatory drugs, tyrosine kinase inhibitors, and anthracycline-based regimens, can also be considered in cases of disease progression for some patient profiles [39] as a potential alternative to the surgical intervention. Although generally limited efficacy has been shown for hormonal agents and nonsteroidal anti-inflammatory drugs.

Despite of the availability of these medical therapies, the present analysis was focused on the comparison of percutaneous cryoablation versus conventional surgery as preliminary to other comparisons which could be further developed.

The present study is a pioneer in the evaluation of percutaneous cryoablation costs in DTs, worldwide and therefore specifically for Spain, also. Previous studies have described the cost of percutaneous cryoablation, as well as its cost-effectiveness compared to other interventions or its budgetary impact, in other regions and other pathologies [40–42]. For the treatment of DTs, economic evaluations of other health technologies have been performed, such as oral treatment with the tyrosine multikinase inhibitor sorafenib [27].

Medical decisions should primarily consider recommendations stated on specific guidelines, ideally supported on robust clinical evidence, as well as individual patients’ needs. However, other criteria, such as an economic profile of therapeutic measures, could add value to this decision-making process, along with contributing to health system sustainability. It should not be forgotten that a drawback of ablation techniques could be that no histopathological analysis is performed, making it impossible to know some tumour subtypes that may be related to the patient's prognosis or could require additional treatment.

Among the main strengths of this study is the validation of the parameters used in the analysis by a panel of clinical experts with extensive experience in the treatment of DTs and, more specifically, in the techniques of interest, which confers good robustness to the study. But the current study has also certain limitations. First, when determining the target population, some estimates have been made based on the opinions and experience of experts. To obtain more accurate figures, it would be better to have a case registry at the national level with a high participation and representation of Spanish hospitals to enable greater monitoring, which in turn would allow continuous learning about the management of DTs. In addition, given the lack of clinical guidelines at the national and/or international level that standardise the management, treatment, and follow-up of DTs, there is a notable variability in clinical practice between hospitals and regions with regards to the specialists involved in the decision making of how to address these tumours and the therapeutic alternatives to be considered. Therefore, this can make it difficult to conduct an analysis for an entire national territory. Moreover, the cost of surgery has been obtained through an indirect calculation by weighting the cases of DTs from four Spanish hospitals and the unit cost of each associated APR-DRG, given the lack of publications with data on costs of surgery. In the event that the aforementioned national case registry is launched in the near future, the cost of surgery could also be calculated with greater precision.

Some bias could be related to the selection of cases based on DRG, so some of the identified codes (93, 98 and 532) seem to be erroneous codifications. Deletion of these cases would yield an average cost for conventional surgery of € 5411. The results which would be derived from this new cost are covered by the range (± 10%) tested on sensitivity analyses performed around this parameter.

Conscious sedation and local anaesthesia instead of general anaesthesia were considered according to the opinion of the experts, as described in recent publications [43], but this could be not totally representative of other settings, where general anaesthesia will be the preferred option [28].

Based on the available evidence [15], a conservative approach was adopted and both techniques were considered comparable in terms of relapses, from first local recurrence and long-term disease control. Therefore, only those costs related to surgical complications were included. However, costs associated to the management of potential relapses could be a source of additional differences in the total cost of the assessed alternatives.

Lastly, the percutaneous cryoablation cost was estimated by considering one of the three cryoablation devices commercially available in Spain, which may represent an additional source of bias, and therefore the results could not be extended or generalised. However, given that the selected device is the most implanted system of percutaneous cryoablation in Spain, the present results represent the most plausible current scenario in the daily practice. Moreover, the estimated cost of percutaneous cryoablation could differ in other settings, due to the legislation about argon storage, but it is not expected to have a great impact on results.

Further future studies could be designed for research about the real resource consumption and relapse rate in patients undergoing percutaneous cryoablation or conventional surgery, avoiding the use of aggregated data as DRG cost and providing reliable data to reassess the present cost analysis.

## Conclusions

Percutaneous cryoablation could lead to cost savings at a hospital level compared to conventional surgery in Spain, as has been shown in this study. In future analyses, it would be beneficial to incorporate the evaluation of the health outcomes of the interventions, as well as to make comparisons with other procedures or expand the analysis to other musculoskeletal tumours.

## Data Availability

All data generated or analysed during this study are included in this published article.

## Abbreviations

APR-DRG: All Patient Refined Diagnosis Related Groups
DRG: Diagnosis-Related Group
DTs: Desmoid tumours
DTWG: Desmoid Tumour Working Group
NHS: National Health System
